# A multi-program analysis of cleft lip with cleft palate prevalence and mortality using data from 22 International Clearinghouse for Birth Defects Surveillance and Research programs, 1974–2014

**DOI:** 10.1002/bdr2.2176

**Published:** 2023-04-26

**Authors:** Niall Mc Goldrick, Gavin Revie, Boris Groisman, Paula Hurtado-Villa, Antonin Sipek, Babak Khoshnood, Anke Rissmann, Saeed Dastgiri, Danielle Landau, Giovanna Tagliabue, Anna Pierini, Miriam Gatt, Osvaldo M. Mutchinick, Laura Martínez, Hermein E.K. de Walle, Elena Szabova, Jorge Lopez Camelo, ECEMC Peripheral Group, Karin Källén, Margery Morgan, Wladimir Wertelecki, Amy Nance, Erin B. Stallings, Wendy N. Nembhard, Peter Mossey

**Affiliations:** 1 School of Dentistry, University of Dundee, Dundee, Scotland, UK; 2 National Network of Congenital Anomalies of Argentina (RENAC), National Institute of Epidemiology (INE), ANLIS, National Ministry of Health, National Center of Medical Genetics, Ciudad de Buenos Aires, Argentina; 3 Facultad de Ciencias de la Salud, Pontificia Universidad Javeriana, Cali, Colombia; 4 Department of Medical Genetics, Thomayer University Hospital, Prague, Czech Republic; 5 Université Paris Cité, Inserm, INRAE, Centre for Research in Epidemiology and StatisticS (CRESS), Obstetrical Perinatal and Pediatric Epidemiology Research Team, EPOPé, Paris, France; 6 Malformation Monitoring Centre Saxony-Anhalt, Medical Faculty Otto-von-Guericke-University Magdeburg, Magdeburg, Germany; 7 Tabriz Health Services Management Research Center, Tabriz University of Medical Sciences, Tabriz, Iran; 8 Israel Birth Defects Surveillance Program, NICU Soroka University Medical Center, Beer Sheva, Israel; 9 Cancer Registry Unit, Fondazione IRCCS Istituto Nazionale dei Tumori, Lombardy birth defects registry, Lombardy, Italy; 10 Institute of Clinical Physiology, National Research Council and Fondazione Toscana Gabriele Monasterio, Tuscany Registry of Congenital Defects, Pisa, Italy; 11 Malta Congenital Anomalies Registry, Directorate for Health Information and Research, Tal-Pietà, Malta; 12 Registry and Epidemiological Surveillance of Congenital Malformations (RYVEMCE), National Institute of Medical Sciences and Nutrition, Mexico City, Mexico; 13 Genetics Department University Hospital, Universidad Autonoma de Nuevo Leon, Nuevo Leon, Mexico; 14 Department of Genetics, University Medical Center Groningen, University of Groningen, Groningen, the Netherlands; 15 Faculty of Public Health, Slovak Medical University, Bratislava, Slovakia; 16 ECLAMC: The Latin-American collaborative study of congenital malformations, Centro de Educacion Médica e Investigaciones Clínicas (CEMIC-CONICET), Buenos Aires, Argentina; 17 Institute of Clinical Sciences, Lund University and National Board of Health and Welfare, Lund, Sweden; 18 Congenital anomaly register and information service, Public Health Wales, Cardiff, Wales, UK; 19 OMNI-Net Ukraine Birth Defects Program, Rivne, Ukraine; 20 Utah Department of Health, Utah Birth Defect Network, Salt Lake City, Utah, USA; 21 Division of Birth Defects and Infant Disorders, National Center on Birth Defects and Development Disabilities, US Centers for Disease Control and Prevention, Atlanta, Georgia, USA; 22 Department of Epidemiology and The Arkansas Reproductive Health Monitoring System, University of Arkansas for Medical Sciences, Little Rock, Arkansas, USA

**Keywords:** craniofacial abnormalities, congenital anomalies, mortality, prevalence, surveillance

## Abstract

**Background:**

Cleft lip with cleft palate (CLP) is a congenital condition that affects both the oral cavity and the lips. This study estimated the prevalence and mortality of CLP using surveillance data collected from birth defect registries around the world.

**Methods:**

Data from 22 population- and hospital-based surveillance programs affiliated with the International Clearinghouse for Birth Defects Surveillance and Research (ICBDSR) in 18 countries on live births (LB), stillbirths (SB), and elective terminations of pregnancy for fetal anomaly (ETOPFA) for CLP from 1974 to 2014 were analyzed. Prevalence and survival (survival for LB only) estimates were calculated for total and subclassifications of CLP and by pregnancy outcome.

**Results:**

The pooled prevalence of total CLP cases was 6.4 CLP per 10,000 births. The prevalence of CLP and all of the pregnancy outcomes varied across programs. Higher ETOPFA rates were recorded in most European programs compared to programs in other continents. In programs reporting low ETOPFA rates or where there was no ascertainment of ETOPFA, the rate of CLP among LB and SB was higher compared to those where ETOPFA rates were ascertained. Overall survival for total CLP was 91%. For isolated CLP, the survival was 97.7%. CLP associated with multiple congenital anomalies had an overall survival of 77.1%, and for CLP associated with genetic/chromosomal syndromes, overall survival was 40.9%.

**Conclusions:**

Total CLP prevalence reported in this study is lower than estimates from prior studies, with variation by pregnancy outcomes between programs. Survival was lower when CLP was associated with other congenital anomalies or syndromes compared to isolated CLP.

## 1 | INTRODUCTION

Cleft lip with cleft palate (CLP) describes a congenital condition that affects both the oral cavity and the lips (Kadir et al., 2017). The condition is a result of the failure of the left and right palatal shelves and lips fusing during the first 9 weeks of fetal development (Berkowitz, 2013). CLP can arise as part of a syndrome or as an isolated disorder and the causes behind CLP are thought to be due to a range of both genetic and environmental factors (Berkowitz, 2013; Cobourne & Sharpe, 2012). The degree of clefting varies from case to case and does not affect each person equally. This article focuses on undifferentiated CLP for which current estimates of prevalence are 1.7 per 1,000 live births (LB) (Mossey, Little, Munger, Dixon, & Shaw, 2009).

The prevalence data available for orofacial clefts (OFC) vary internationally due to differences in ascertainment ability, registry resources, and comparability of the conditions classified in reported studies. A European study carried out across 17 different nations demonstrated variation between 6.3 and 26.2 per 10,000 births for all orofacial clefts (cleft palate or cleft lip +/− cleft palate) (mean prevalence 15.2 per 10,000 births) (Calzolari, Rubies, Neville, & Bianchi, 2002). As with many conditions, high-income countries have a greater ability to conduct birth defect surveillance due to more advanced health systems and centrally organized registries (Swanson, 2021). In low- and middle- income countries, the resources available for birth defect surveillance are reduced, which impacts data availability and prevents accurate international comparisons and inferences (Cobourne & Sharpe, 2012).

Mortality of infants born with OFC is associated with the lack of access to appropriate care and surgical intervention (Cobourne & Sharpe, 2012). The diagnosis and treatment available for children with OFC varies internationally, leading to inequalities in health outcomes (Mossey et al., 2009). Understanding where mortality rates are high could help to target further research and interventions to reduce mortality, improve quality of life, and provide greater equity of care. Prevalence data from multiple countries would guide future research to identify risk factors, policies, or ascertainment methods that give rise to variation globally, including nutrition/ fortification policies, policies regarding early termination of pregnancy for fetal anomaly ETOPFA, prenatal care arrangement, and prevalence of underlying genetic/chromosomal anomalies in the parent population. Understanding more about these associations could enable development and testing of preventative interventions.

The International Clearinghouse for Birth Defects Surveillance and Research (ICBDSR) was founded in 1974 and is affiliated with the World Health Organization. It has a stated mission to “bring together birth defect programs from around the world with the aim of conducting worldwide surveillance and research to prevent birth defects and to ameliorate their consequences” (ICBDSR, n.d.). The ICBDSR includes 42 programs spread across the world with a mixture of population- and hospital-based registries. Data collected by ICBDSR programs enable analysis of the prevalence, pregnancy outcomes, and survival for a range of congenital anomalies on an international basis.

The aim of this retrospective cohort study was to analyze undifferentiated CLP birth surveillance data from participating ICBDSR programs to estimate the prevalence and survival of CLP by pregnancy outcomes while identifying areas for improvement in data collection processes for this type of study.

## 2 | METHODS

The structure and content of this article is informed by The Strengthening the Reporting of Observational Studies in Epidemiology (STROBE) Statement: guidelines for reporting observational studies (von Elm et al., 2007).

### 2.1 | Case definition

The primary congenital anomaly reported in this study is undifferentiated CLP. This includes all CLP cases including isolated CLP (no other orofacial anomalies identified), CLP associated with multiple congenital anomalies, and CLP associated with syndromes. Isolated cleft palate and isolated cleft lip have been reported separately to this data set and are, therefore, not included here. Cases included all identified conceptions, resulting in an individual with CLP, regardless of outcome. Where data were available, subclassifications including isolated CLP, CLP associated with multiple congenital anomalies, and CLP associated with syndromes are reported as mutually exclusive categories.

Keeping with accepted terminology, birth prevalence is used in this article to describe the point prevalence of CLP in discrete populations included in ICBDSR programs (Mason, Kirby, Sever, & Langlois, 2005). Mason et al. (2005) suggested using total births alone as the denominator, but those data were not available in this study, so a slightly modified equation was used. Birth prevalence was calculated as follows:

$$Birth\;prevalence=\frac{Number\;of\;cases}{Total\;Live\;births\;+\;Total\;Stillbirths}.$$

### 2.2 | Data source

All ICBDSR programs were invited to participate. Twenty-three birth surveillance programs from 18 countries provided data covering a range of time periods within the date range 1974–2014. Each program returned a single data set except for the Registry of the Spanish Collaborative Study of Congenital Malformations (ECEMC). ECEMC provided data from two different hospital cohorts, one reporting ETOPFA and the other where data regarding ETOPFA was explicitly not recorded. The ECEMC data sets have been treated separately in the data analysis.

The programs are a mix of population and hospital-based registries. Raw data were provided in the form of MS Excel documents with cases classified by pattern into the following categories: isolated CLP, CLP associated with multiple congenital anomalies, CLP associated with syndromes, and CLP unclassified. No program returned data for CLP unclassified and, therefore, analysis is focused on the other categories only. Further to this, all programs reported a “Total CLP” value that combines all the aforementioned subclassifications.

Data sets included varying amounts of data on LB, stillbirths (SB) and ETOPFA for each of the CLP subclassifications. The most complete data set across all programs was ‘Total CLP’ and this was selected for more detailed analysis with descriptive statistics presented for subclassifications where possible.

### 2.3 | Data quality assessment/Data analysis

Data were extracted and combined using the Microsoft Excel. Primary inspection and analysis of the data were conducted using R (R Core Team, 2021). The data were inspected and cleaned. Data anomalies identified in the reporting triggered dialog with the reporting programs for clarification and correction of errors where possible. Data quality issues were considered by NMG, GR, and PM following early data cleaning. Data sets with overwhelming data errors or omissions following attempts to clarify were excluded from the analysis (*n* = 1). Therefore, data from 22 surveillance programs amounting to 23 data sets have been included in the analysis.

The quantities of data and formatting of the data files varied considerably between programs, so automated importation was not practical and the data had to be manually imported one file at a time. The data set from each program was individually copied and pasted into a large Excel “master” file, which was of a format suitable for analysis in R. The data were inspected primarily through the use of the aggregate command and ggplot2 to produce summary statistic tables and graphs. Where possible, the data were further checked for obvious errors (e.g., extremely low or high prevalence or mathematical errors such as more deaths than reported cases in a given year).

Not all programs provided data for the entire observation period; therefore, data provided were averaged for the period that each program provided results. For example, when calculating prevalence, while the number of years returned varied, the denominator in all cases was the total number of LB plus SB reported by that program for all the years they returned data, and the numerator was the total cases observed during that same period.

Survival was calculated using data for LB only. Survival data are presented as percentages surviving at timepoints from <1 day to 5 years + where this was ascertained. Overall survival includes the timepoint survival data and any death confirmed but without a timepoint attached.

### 2.4 | Ethical consideration

ICBDSR programs providing data for this study have done so according to local ethical procedures and review. Only aggregated data without any personal identifiers were used in this study, and therefore, further ethical review was not required.

## 3 | RESULTS

Data from 22 programs amounting to 23 separate data sets were included in the analysis. This included a total number of 23,523,031 births and 15,103 CLP cases. Table 1 provides a description (location, type of registry, area covered, ascertainment period, stillbirth definition, whether ETOPFA is permitted, and prenatal screening services) of ICBDSR programs providing data and included in this study. A description of the follow-up method for LB for each program is presented in Table 2.

Table 3 presents descriptive statistics including pregnancy outcomes for each of the included programs (total number of births, total number of CLP cases, prevalence per 10,000 births, percentage of LB among CLP cases, percentage of SB among CLP cases, and percentage of ETOPFA among CLP cases) for the observation period 1974–2014. Tables 4, 5, and 6 present similar descriptive statistics for programs that provided data for each of the subclassifications of CLP described in the methods.

**FIGURE 1:**
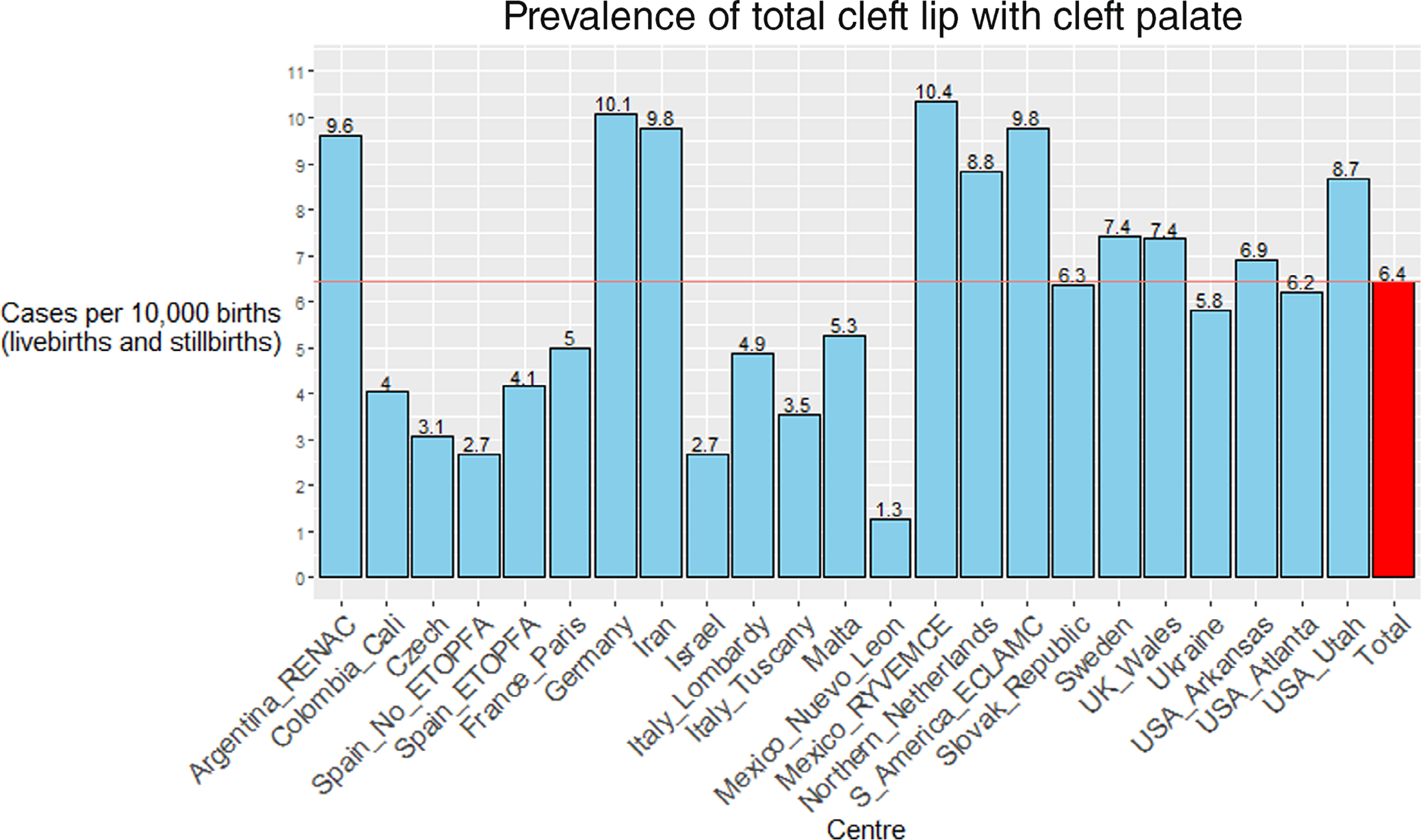
Pooled prevalence per 10,000 births of total cleft lip with the cleft palate for each participating ICBDSR program for the period 1974–2014.

The prevalence of total CLP for each of the included programs ranged from 1.3 per 10,000 births (Mexico Neuvo Leon) to 10.4 per 10,000 births (Mexico RYVEMCE) and is presented in Figure 1. The pooled average for total CLP prevalence from all programs across the observation period was 6.4 per 10,000 births.

**FIGURE 2:**
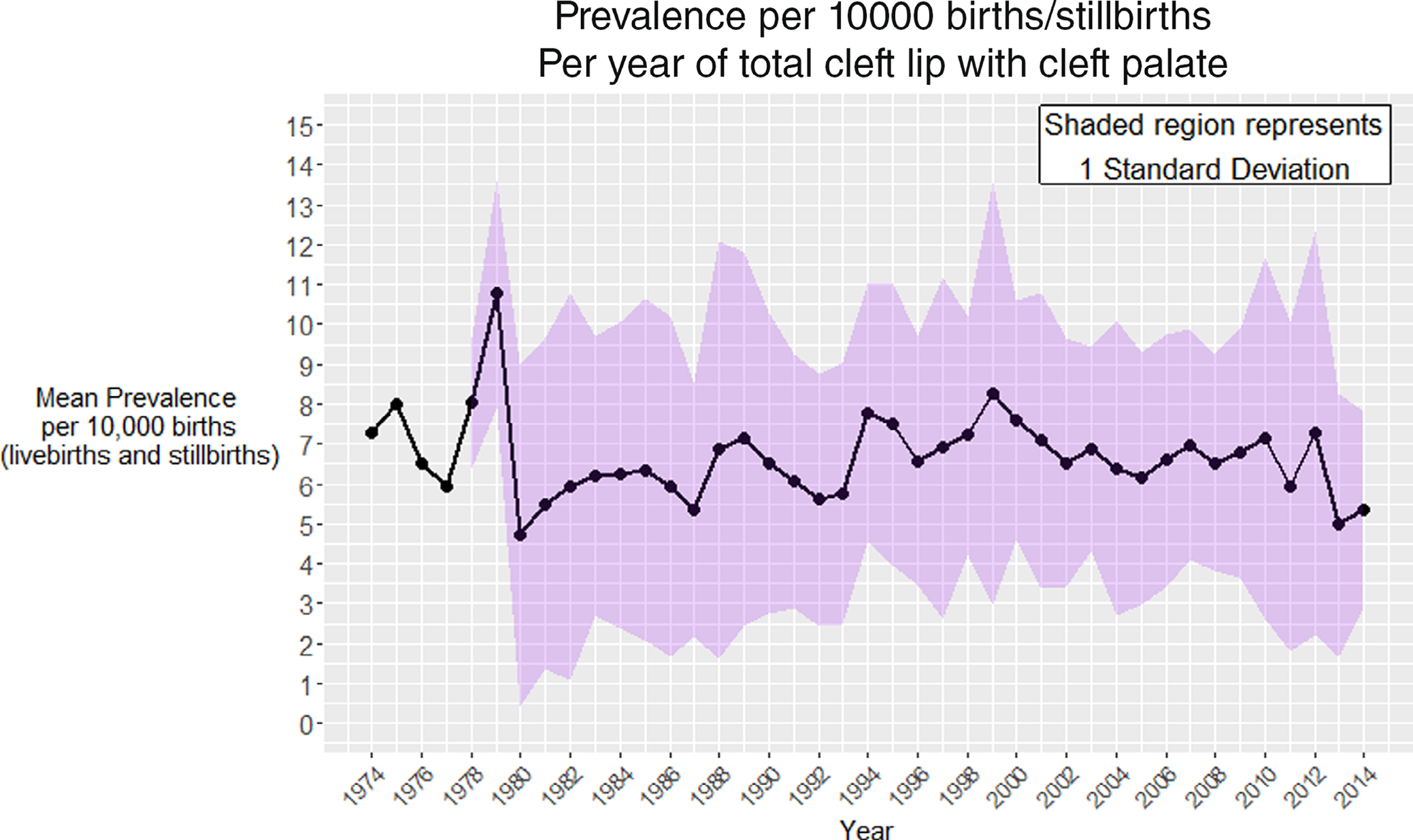
Prevalence by year of undifferentiated cleft lip with the cleft palate.

The mean prevalence of total CLP varied each year with a maximum of 11 per 10,000 births (live and still births) reported in 1979 followed by a range between 4.5 per 10,000 births (live and still births) to 8.5 per 10,000 births (live and still births). There is significant spread of data around the mean, illustrated in Figure 2.

The survival rates of LB varied across programs. A description of the percentage of LB surviving at timepoints varying from 1 day through 5 years for total CLP followed by each of the subclassifications is presented in Table 7. Overall survival is also presented for each program. The pooled average of surviving LB for total CLP was 91% when considering all-cause mortality. The pooled average for isolated CLP was 97.7%. For CLP associated with multiple congenital anomalies, the average surviving LB once all-cause mortality was considered was 77.1%, and for CLP associated with genetic or chromosomal syndromes was 40.9%.

## 4 | DISCUSSION

Strengths and limitations of this study are discussed throughout this section. The prevalence of total CLP varied substantially across programs ranging from 1.26 to 10.37 per 10,000 births for the observation period. Surveillance methods and ascertainment of the presence of clefts varied during the observation period and between programs. Important differences include hospital- versus population-based registries with hospital programs serving a select sample of a wider population. The geographical area covered by the program is also important to consider, as areas with a local registry may be skewed by local clusters, although this can be very useful when aiming to identify possible causes for perceived higher prevalence associated with a program that may be due to local environmental or genetic influences. Analyses in this study do not account for the heterogeneity between programs. The context of the country, culture, and health system where each registry is based should be taken into account when interpreting the data presented in this article. While these data may point to further questions related to causality, it is not possible to draw inferences on causality from these data. The data will provide utility to reporting programs to discuss and interpret locally.

The pooled birth prevalence presented for total CLP of 6.4 per 10,000 births is slightly lower than what would have been expected from the global literature. Mossey et al. presented a prevalence of 1 per 700 LB corresponding to 14.3 per 10,000 LB for all OFC and Massenburg present a prevalence of 141.56 per 100,000 of OFC in their study population corresponding to 14.1 per 10,000 LB (Massenburg et al., 2021; Mossey et al., 2009). In Europe, a reported prevalence of 14.26 OFC per 10,000 births is reported (European Commission, 2022). Considering that CLP makes up around 50% of the OFC cases, our prevalence estimate from these ICBDSR data is slightly lower than the average global CLP prevalence of around 7.0 per 10,000 (Fogh-Anderson, 1942; Mossey P.A., 2002). The study by the European Commission reported no significant findings in trends over time during a 26-year observation period (European Commission, 2022). Swanson described some of the issues associated with comparability across orofacial cleft epidemiology studies, including data availability and ascertainment (Swanson, 2021). The variability in case definition among published studies makes direct comparison difficult. A range of factors may contribute to the low prevalence reported in our study, such as the inclusion of SB in the denominator data, which may impact the prevalence calculation, variance in the ability of programs to ascertain all cases, and variance in the source used for denominator populations between hospital-based or regional programs.

The rate of ETOPFA for total CLP in most European programs was higher than other continents with notable exceptions such as Malta, where termination of pregnancy is not legal (including for anomalies that are fatal beyond the womb), and the reported rate of ETOPFA was 0% for that surveillance program. A 19% stillbirth rate for total CLP was reported for Malta; it is important to note that the SB were related to chromosomal syndromes and multiple congenital abnormality cases, and the total number of CLP cases for Malta was small, 42 cases, highlighting the need to apply caution when comparing programs. Previous reports of ETOPFA with CLP among European populations averaged 11.8% (Calzolari et al., 2002). A further program of note is Israel, where terminations or SB available but are not registered. For programs that provided data on the subclassifications, the percentage of ETOPFA for isolated CLP was low when compared to the other subclassifications for CLP associated with multiple congenital anomalies and CLP associated with genetic/chromosomal syndrome. This is a positive finding for isolated CLP as this birth anomaly can be surgically repaired resulting in effective cure for the majority of cases (Williams et al., 2001). Unfortunately, access to quality surgical care is not universal, as demonstrated by a 2015 Lancet Commission (Meara et al., 2015). The low rates of EOPTFA for isolated clefts are similar to that reported in other studies (Calzolari et al., 2002; Yazdy, Honein, & Xing, 2007).

Identification of variations in survival is an essential component of the Global Burden of Disease (GBD) project for monitoring progress in global health and alleviation by access to care (Horton, 2012). The variation in the overall survival of LB for total CLP presented in Table 7 shows a tendency for higher survival rates among programs in Europe but with some exceptions such as Malta (where termination of pregnancy is illegal) with an overall survival of 85% compared to the cohort average of 91%. The survival data for the subclassifications demonstrate clear differences among subclassifications with isolated CLP cases having the highest rates of survival. Issues surrounding infant mortality in the presence of birth defects are important in the context of primary prevention, and in the case of CLP, timely access to primary cleft repair results in survival rates equivalent to unaffected infants (Christensen, Juel, Herskind, & Murray, 2004; Cobourne & Sharpe, 2012). Christensen et al. demonstrated an increase in mortality among CLP cases compared to standardized rates in a Danish population (Christensen et al., 2004). Mossey and Modell (2002) have explored the influence of access to care on survival, finding that access to comprehensive (multidisciplinary) cleft care coincides with improved survival; most programs in this study are in countries providing comprehensive care (Cobourne & Sharpe, 2012). Furthermore, cleft lip repair has been suggested as a marker of the provision of essential pediatric care, particularly in low- and middle-income countries (Vanderburg et al., 2021). It is important to note the majority of programs (64%) included in this study are from countries that are classified as high-income by the World Bank. Further consideration of efforts to improve ascertainment and recording of CLP cases in low- and low-middle-income countries should be pursued.

As has been reported in other studies of this kind, some of the variation in findings may be due, in part, to variation in ascertainment, available diagnostics, and data quality issues associated with data submitted for this study; therefore, the results should be interpreted with caution reflecting on the limitations described above (Calzolari et al., 2002; Cobourne & Sharpe, 2012). A detailed diagnostic document has been produced exploring the source data used for this article (Revie, 2020). This presents a number of areas for improvement in data extraction and processes to standardize the approach within reporting programs internationally. It is also suggested to add certain information to the data collection that would aid in useful comparison data across programs and allow for further useful statistical analysis, for example, calculation of risk ratios for specific outcomes.

**FIGURE 3:**
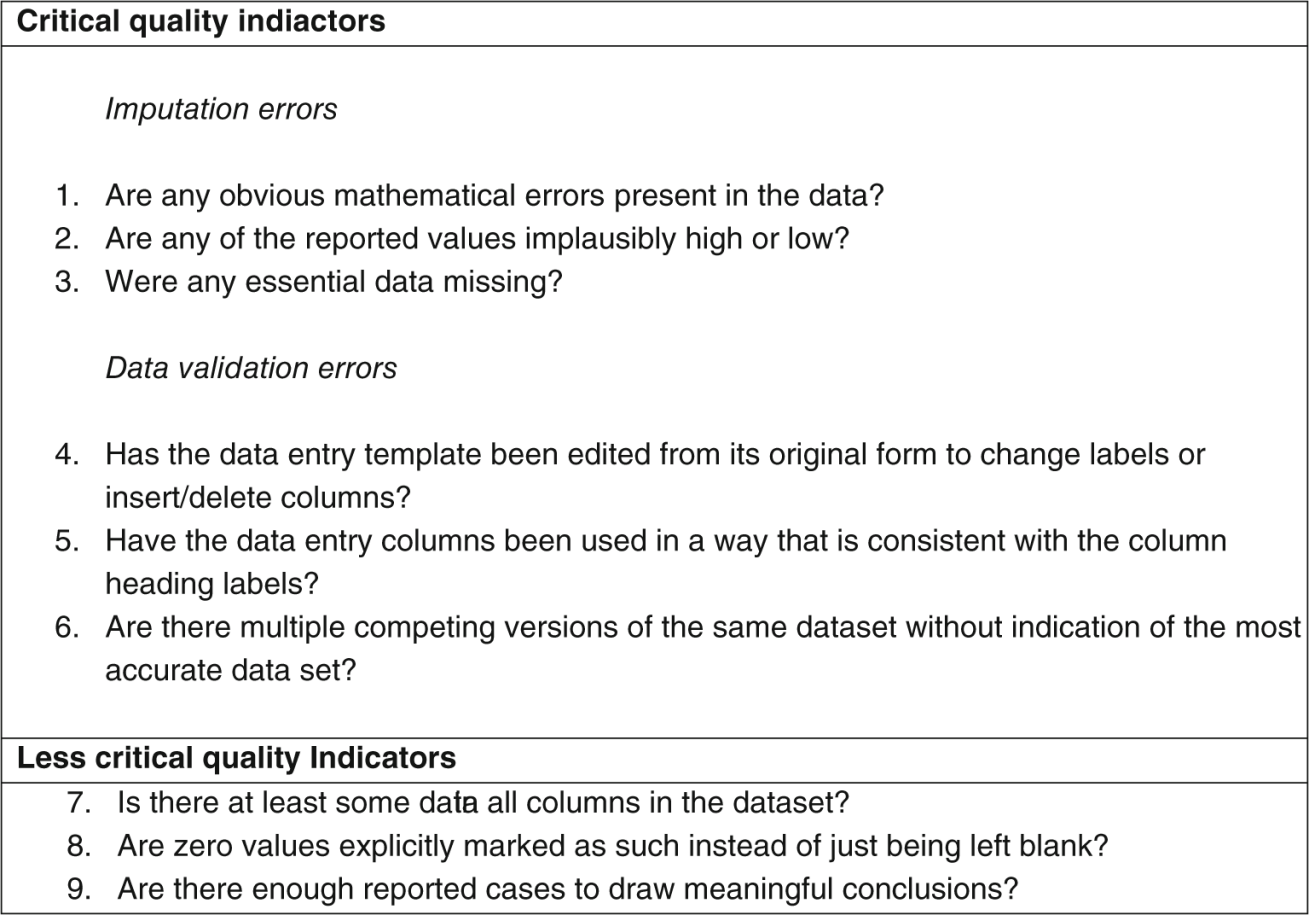
Quality indicators used by authors to assess data quality of data sets and inform inclusion criteria.

Following discussion and further consideration the authors propose a draft set of data quality indicators that may improve data quality in future studies of this kind (Figure 3). The indicators reflect some of the analytical and data management issues presented with this large and complex set of data. Factors are separated into “critical” and “less critical”. Critical missing data and evident calculation errors are given a heavier weighting, whereas empty cells in otherwise complete data sets receive a lower weighting. The draft quality indicators and the detailed diagnostic analysis document (not published) may form the basis for discussion and future work to improve the quality of the data extracted and reported from programs. A working group focused on standardizing data collection forms, guidance, and regular reporting mechanisms for CLP may prove beneficial enabling an improvement in the validity and reliability of similar data sets in the future.

Adopting a health economics approach and incorporating this in future analysis of orofacial clefts and other congenital anomalies should be considered. Similar analysis that includes the suggestions above could be applied to other data sets held by ICBDSR, including the cleft palate with and without Robin sequence and cleft lip without the cleft palate.

## 5 | CONCLUSION

Total CLP prevalence of 6.4 per 10,000 births as reported in this study is regarded as slightly lower than previous global estimates. There was variation across included programs for prevalence, pregnancy outcomes, and survival. The survival of LB with CLP was greatest among cases with isolated CLP (97.7%) and worst among CLP cases associated with genetic or chromosomal syndromes (40.9%). Data quality and heterogeneity among data sets have both been highlighted in this study, and efforts to improve data quality related to future CLP epidemiology studies deserve consideration.

## Figures and Tables

**TABLE 1:** Description of ICBDSR Birth Surveillance programs providing data and included in this study.

Country	Birth surveillance program	Population or hospital-based program	Area covered Ascertainment period	Ascertainment method^a^	Stillbirth definition	ETOPFA permitted	Prenatal screening services
Argentina	RENAC	Hospital	National Hospital discharge	Passive	>500 g	No	Yes, but no official program
Colombia	Cali	Hospital	Regional 1 day/hospital discharge	Passive	>500 g	Yes, since 2006, but not registered	Yes
Czech Republic	Czech	Population	National 15 years	Passive	22 weeks or >500 g	Yes	Yes
Spain	ECEMC hospitals not reporting ETOPFA	Hospital	Regional 3 days	Active	24 weeks or >500 g	Yes, since 1985	Yes
Spain	ECEMC hospitals reporting ETOPFA	Hospital	Regional 3 days	Active	24 weeks or >500 g	Yes, since 1985	Yes
France	Paris	Population	Regional 28 days	Active	22 weeks or >500 g	Yes	Yes
Germany	Saxony-Anhalt	Population	Regional 1 year	Passive	>500 g	Yes	Yes, since 1990
Iran	Tabriz Registry of Congenital Anomalies (TRoCA)	Hospital	Regional Hospital Discharge	Hybrid	20 weeks or >400 g	Yes, but not formally registered.	Yes
Israel	Israel	Hospital	Regional Hospital discharge	Passive	22 weeks or >500 g	Yes, not registered	Yes
Italy	Lombardy	Population	Regional 6 years	Passive	22 weeks	Yes	Yes
Italy	Tuscany	Population	Regional 1 year	Passive	20 weeks	Yes	Yes
Malta	Malta	Population	National 1 year	Active	22 weeks or >500 g	No	Limited
Mexico	Nuevo Leon	Population	Regional 6 Days	Active	20 weeks	No	Yes
Mexico	RYVEMCE	Hospital	Regional 3 days	Active	20 weeks or >500 g	No	In a small number of institutions but not nationally
Netherlands	Eurocat Northern Netherlands	Population	Regional 10 years	Active	24 weeks	Yes	Yes, since 2007
South America	ECLAMC	Hospital	Regional Hospital discharge	Passive	>500 g	Yes	Yes
Slovak Republic		Population	National Hospital discharge	Passive	>500 g	Yes	Yes
Sweden	Sweden	Population	National Until 1986:1 month, since 1987:1 year	Passive	Until 2006:28 weeks, since 2007:22 weeks	Yes, since 1999	Yes, since early 1980’s
UK	Wales	Population	Regional 18 years	Active	24 weeks	Yes	Yes, since 2003
Ukraine	OMNI-Net BD program	Population	Regional Until 2002:7 days, 2003– 2007:28 days, since 2008–1 year.	Active	Until 2006:28 weeks or >1,000 g; since 2007:22 weeks or >500 g.	Yes	Yes
USA	Arkansas	Population	State 2 years	Active	20 weeks	Yes, up to 20 weeks	Yes
USA	Atlanta	Population	Regional 6 years	Active	20 weeks	Yes	Yes
USA	Utah	Population	State 2 years	Active	20 weeks	Yes	Yes

Abbreviations: ECEMC, Registry of the Spanish Collaborative Study of Congenital Malformations; ECLAMC, Latin American Collaborative Study of Congenital Malformations; ETOPFA, Early termination of pregnancy for fetal anomaly; ICBDSR, International Clearing House for Birth defects Surveillance and Research; RENAC, National Network of Congenital Anomalies of Argentina; RYVEMCE, Mexican Registry and Epidemiological Surveillance of External Congenital Malformations; SMC, Soroka Medical Center; UK, United Kingdom; USA, United States of America.

a Defined using CDC birth defects surveillance toolkit: https://www.cdc.gov/ncbddd/birthdefects/surveillancemanual/facilitators-guide/module-3/mod3-3.html.

**TABLE 2:** Description of the follow-up method for live births from ICBDSR programs contributing to this study.

Country	Birth surveillance program	Until discharge from the maternity hospital	By clinician or program staff	Linkage with death certificates
Argentina	RENAC	Yes	Yes	No
Colombia	Cali	Yes	Yes	No
Czech Republic	Czech	No	No	Yes
Spain	ECEMC hospitals not reporting ETOPFA	Yes	Yes	No
Spain	ECEMC hospitals reporting ETOPFA	Yes	Yes	No
France	Paris	Yes	Yes	No
Germany	Saxony-Anhalt	Yes	Yes	No
Iran	Tabriz Registry of Congenital Anomalies (TRoCA)	Yes	Yes	No
Israel	Israel	Yes	Yes	Yes
Italy	Lombardy	no	yes	Yes, 2003 up to 2021
Italy	Tuscany	No	No	Yes, 1992 up to 2015
Malta	Malta Congenital Anomalies Registry (MCAR)	No	Hospital files followed up until 1 year of age	Yes
Mexico	Nuevo Leon	Yes	Yes	Yes
Mexico	RYVEMCE	Yes	Only until discharge from maternity hospital	No
Netherlands	Eurocat Northern Netherlands	Yes	Yes	No
South America	ECLAMC	Yes	Yes	No
Slovak Republic		Yes	Only until discharge from the maternity hospital	No
Sweden	Sweden	No	No	No
UK	Wales	Yes	Only until discharge from maternity hospital	Yes, up to 18 years
Ukraine	OMNI-Net BD program	Yes	Yes	No
USA	Arkansas	Yes	Only until discharge from maternity hospital	Yes, 1993 up to 2015
USA	Atlanta	Yes	Only until discharge from maternity hospital with abstract of visits to children’s hospitals until age 6.	Yes, 1979 up to 2008
USA	Utah	Yes	Only until discharge from maternity hospital	Yes, until age 2

Abbreviations: ECEMC, Registry of the Spanish Collaborative Study of Congenital Malformations; ECLAMC, Latin American Collaborative Study of Congenital Malformations; ETOPFA, early termination of pregnancy for fetal anomaly; ICBDSR, International Clearing House for Birth defects Surveillance and Research; RENAC, National Network of Congenital Anomalies of Argentina; RYVEMCE, Mexican Registry and Epidemiological Surveillance of External Congenital Malformations; SMC, Soroka Medical Center; UK, United Kingdom; USA, United States of America.

**TABLE 3:** Total CLP by birth outcome during 1974–2014 from reporting ICBDSR programs. Presented in order of prevalence.

Country	Birth surveillance program	Population or hospital- based program	Observation period	Total births^a^	Total CLP cases	Total CLP prevalence per 10,000 births	Total CLP Live birth %	Total CLP stillbirth %	Total CLP ETOPFA (%)
Mexico	RYVEMCE	Hospital	1978–2013	1,198,579	1,243	10.37	90.43	9.57	0
Germany	Saxony-Anhalt	Population	1980–2014	526,289	531	10.09	83.43	6.97	9.6
South America	ECLAMC	Hospital	1995–2014	2,927,555	2,862	9.78	90.64	9.36	0
Iran	Tabriz Registry of Congenital Anomalies (TRoCA)	Hospital	2004–2012	160,755	157	9.77	99.36	0.64	0
Argentina	RENAC	Hospital	2009–2014	1,023,108	983	9.61	97.15	2.85	No data^b^
Netherlands	Eurocat Northern Netherlands	Population	1981–2014	562,462	496	8.82	93.95	2.62	3.43
USA	Utah	Population	1995–2012	889,634	771	8.67	90.66	3.37	5.97
Sweden	Sweden	Population	1974–2014	4,195,523	3,113	7.42	95.47	0.93	3.6
UK	Wales	Population	1998–2014	569,341	419	7.36	80.19	0.95	18.38
USA	Arkansas	Population	1993–2012	760,777	525	6.9	94.86	3.62	0.95
Slovak Republic		Population	2001–2013	722,978	459	6.35	97.39	0.87	1.09
USA	Atlanta	Population	1994–2008	737,250	458	6.21	85.59	5.02	8.52
Ukraine	OMNI-Net BD program	Population	2000–2013	404,172	235	5.81	81.28	2.13	14.47
Malta	Malta Congenital Anomalies Registry (MCAR)	Population	1995–2013	79,948	42	5.25	80.95	19.05	0
France	Paris	Population	1981–2014	875,241	436	4.98	70.64	5.28	24.08
Italy	Lombardy	Population	2003–2012	133,182	65	4.88	80	3.08	16.92
Spain	ECEMC hospitals reporting ETOPFA	Hospital	1995–2013	373,698	155	4.15	65.16	1.29	33.55
Colombia	Cali	Hospital	2011–2014	27,294	11	4.03	100	0	0
Italy	Tuscany	Population	1992–2014	636,562	225	3.53	81.78	2.22	16
Czech Republic	Czech	Population	1980–2014	4,034,194	1,244	3.08	88.1	0.48	11.41
Spain	ECEMC hospitals not reporting ETPOFA	Hospital	1986–2013	2,135,249	575	2.69	96.87	3.13	No data^b^
Israel		Hospital	2000–2014	200,660	54	2.69	100	0	0
Mexico	Nuevo Leon	Population	2011–2014	348,580	44	1.26	100	0	0

Abbreviations: ECEMC, Registry of the Spanish Collaborative Study of Congenital Malformations; ECLAMC, Latin American Collaborative Study of Congenital Malformations; ETOPFA, early termination of pregnancy for fetal anomaly; ICBDSR, International Clearing House for Birth defects Surveillance and Research; RENAC, National Network of Congenital Anomalies of Argentina; RYVEMCE, Mexican Registry and Epidemiological Surveillance of External Congenital Malformations; SMC, Soroka Medical Center; UK, United Kingdom; USA, United States of America.

a Live births and still births.

b No data for this measure were confirmed by the registry.

**TABLE 4:** Isolated CLP by birth outcome during 1974–2014 from reporting ICBDSR programs. Presented in order of prevalence.

Country	Birth surveillance program	Population or hospital-based program	Observation period	Total Births^a^	Isolated CLP cases	Isolated CLP prevalence per 10,000 births	Isolated CLP Live birth %	Isolated CLP Stillbirth %	Isolated CLP ETOPFA(%)
Mexico	RYVEMCE	Hospital	1978–2013	1,198,579	912	7.61	96.27	3.73	0
Germany	Saxony-Anhalt	Population	1980–2014	526,289	393	7.47	94.4	4.58	1.02
Netherlands	Eurocat Northern Netherlands	Population	1981–2014	562,462	398	7.08	98.24	1.26	0.5
Argentina	RENAC	Hospital	2009–2014	1,023,108	701	6.85	99.29	0.71	No data^b^
USA	Utah	Population	1995–2012	889,634	534	6	95.69	1.5	2.81
South America	ECLAMC	Hospital	1995–2014	2,927,555	1727	5.9	96.87	3.13	0
Sweden	Sweden	Population	1974–2014	4,195,523	2,461	5.87	99.02	0.57	0.41
Slovak Republic		Population	2001–2013	722,978	351	4.85	99.15	0.85	0
UK	Wales	Population	1998–2014	569,341	257	4.51	98.44	0	1.56
Ukraine	OMNI-Net BD Program	Population	2000–2013	404,172	170	4.21	91.76	0.59	6.47
Colombia	Cali	Hospital	2011–2014	27,294	11	4.03	100	0	0
France	Paris	Population	1981–2014	875,241	267	3.05	95.13	1.5	3.37
Italy	Lombardy	Population	2003–2012	133,182	38	2.85	97.37	2.63	0
Malta	MCAR	Population	1995–2013	79,948	20	2.5	100	0	0
Israel		Hospital	2000–2014	200,660	45	2.24	100	0	0
Spain	ECEMC hospitals reporting ETOPFA	Hospital	1995–2013	373,698	83	2.22	92.77	0	7.23
Spain	ECEMC hospitals not reporting ETOPFA	Hospital	1986–2013	2,135,249	393	1.84	98.73	1.27	No data^b^

Abbreviations: ECEMC, Registry of the Spanish Collaborative Study of Congenital Malformations; ECLAMC, Latin American Collaborative Study of Congenital Malformations; ETOPFA, early termination of pregnancy for fetal anomaly; ICBDSR, International Clearing House for Birth defects Surveillance and Research; RENAC, National Network of Congenital Anomalies of Argentina; RYVEMCE, Mexican Registry and Epidemiological Surveillance of External Congenital Malformations; SMC, Soroka Medical Center; UK, United Kingdom; USA, United States of America.

a Live births and still births.

b No data for this measure were confirmed by the registry.

**TABLE 5:** CLP cases associated with multiple congenital anomalies by birth outcome during 1974–2014 from reporting ICBDSR programs. Presented in order of prevalence.

Country	Birth surveillance program	Population or hospital-based program	Observation period	Total Births^a^	CLP with multiple congenital anomalies cases	CLP with multiple congenital anomalies prevalence per 10,000 births	CLP with multiple congenital anomalies Live birth %	CLP with multiple congenital anomalies Stillbirth %	CLP with multiple congenital anomalies ETOPFA(%)
South America	ECLAMC	Hospital	1995–2014	2,927,555	1,135	3.88	81.15	18.85	0
Argentina	RENAC	Hospital	2009–2014	1,023,108	251	2.45	92.43	7.57	No data^b^
Italy	Lombardy	Population	2003–2012	133,182	27	2.03	55.56	3.7	40.74
Germany	Saxony-Anhalt	Population	1980–2014	526,289	93	1.77	60.22	13.98	25.81
Malta	MCAR	Population	1995–2013	79,948	14	1.75	78.57	21.43	0
Mexico	RYVEMCE	Hospital	1978–2013	1,198,579	194	1.62	72.68	27.32	0
UK	Wales	Population	1998–2014	569,341	90	1.58	61.11	4.44	34.44
USA	Utah	Population	1995–2012	889,634	132	1.48	87.88	3.79	8.33
Slovak Republic		Population	2001–2013	722,978	95	1.31	94.74	1.05	4.21
Spain	ECEMC hospitals reporting ETOPFA	Hospital	1995–2013	373,698	44	1.18	45.45	2.27	52.27
Ukraine	OMNI-Net BD Program	Population	2000–2013	404,172	47	1.16	51.06	4.26	40.43
France	Paris	Population	1981–2014	875,241	92	1.05	40.22	16.3	43.48
Sweden	Sweden	Population	1974–2014	4,195,523	387	0.92	85.53	2.33	12.14
Netherlands	Eurocat Northern Netherlands	Population	1981–2014	562,462	40	0.71	87.5	10	2.5
Spain	ECEMC hospitals not reporting ETOPFA	Hospital	1986–2013	2,135,249	118	0.55	93.22	6.78	No data^b^
Israel		Hospital	2000–2014	200,660	9	0.45	100	0	0

Abbreviations: ECEMC, Registry of the Spanish Collaborative Study of Congenital Malformations; ECLAMC, Latin American Collaborative Study of Congenital Malformations; ETOPFA, early termination of pregnancy for fetal anomaly; ICBDSR, International Clearing House for Birth defects Surveillance and Research; RENAC, National Network of Congenital Anomalies of Argentina; RYVEMCE, Mexican Registry and Epidemiological Surveillance of External Congenital Malformations; SMC, Soroka Medical Center; UK, United Kingdom; USA, United States of America.

a Live births and still births.

b No data for this measure were confirmed by the registry.

**TABLE 6:** Cases CLP with genetic or chromosomal syndromes by birth outcome during 1974–2014 from reporting ICBDSR programs. Presented in order of prevalence.

Country	Birth surveillance program	Population or hospital- based program	Observation period	Total births^a^	CLP with genetic or chromosomal syndrome cases	CLP with genetic or chromosomal syndrome prevalence per 10,000 births	CLP with genetic or chromosomal syndrome Live birth %	CLP wdth genetic or chromosomal syndrome Stillbirth %	CLP wdth genetic or chromosomal syndrome ETOPFA(%)
UK	Wales	Population	1998–2014	569,341	70	1.23	40	0	60
USA	Utah	Population	1995–2012	889,634	105	1.18	68.57	12.38	19.05
Mexico	RYVEMCE	Hospital	1978–2013	1,198,579	137	1.14	76.64	23.36	0
Netherlands	Eurocat Northern Netherlands	Population	1981–2014	562,462	58	1.03	68.97	6.9	24.14
Malta		Population	1995–2013	79,948	8	1.00	37.5	62.5	0
France	Paris	Population	1981–2014	875,241	77	0.88	22.08	5.19	72.73
Germany	Saxony-Anhalt	Population	1980–2014	526,289	45	0.86	35.56	13.33	51.11
Spain	ECEMC hospitals reporting ETOPFA	Hospital	1995–2013	373,698	28	0.75	14.29	3.57	82.14
Sweden	Sweden	Population	1974–2014	4,195,523	265	0.63	76.98	2.26	20.75
Italy	Lombardy	Population	2003–2012	133,182	7	0.53	14.29	14.29	71.43
Ukraine	OMNI-Net BD Program	Population	2000–2013	404,172	18	0.45	61.11	11.11	22.22
Argentina	RENAC	Hospital	2009–2014	1,023,108	31	0.3	87.1	12.9	No data^b^
Spain	ECEMC hospitals not reporting ETOPFA	Hospital	1986–2013	2,135,249	64	0.3	92.19	7.81	No data^b^
Slovak Republic		Population	2001–2013	722,978	14	0.19	92.86	0	7.14
Czech Republic	Czech	Population	1980–2014	4,034,194	49	0.12	67.35	0	32.65

Abbreviations: ECEMC, Registry of the Spanish Collaborative Study of Congenital Malformations; ECLAMC, Latin American Collaborative Study of Congenital Malformations; ETOPFA, early termination of pregnancy for fetal anomaly; ICBDSR, International Clearing House for Birth defects Surveillance and Research; RENAC, National Network of Congenital Anomalies of Argentina; RYVEMCE, Mexican Registry and Epidemiological Surveillance of External Congenital Malformations; SMC, Soroka Medical Center; UK, United Kingdom; USA, United States of America.

a Live births and still births.

b No data for this measure were confirmed by the registry.

**TABLE 7:** Percentage of live births surviving at various timepoints from day 1 to 5 years including overall survival. Data are presented for total CLP for all 22 reporting ICBDSR programs and data for subclassifications where available.

				Percentage surviving						
Sub-classification	Region	Center	Live births Count	Day <1	Days 1–6	Days 7–27	28 Days–11 Months	Years 1–4	5 years +	Overall survival
Total	Spain	ECEMC hospitals reporting ETOPFA	101	99	99	99	99	99	99	99
	Iran		156	98.7	98.1	98.1	98.1	98.1	98.1	98.1
	Germany	Saxony-Anhalt	443	99.1	98	97.1	96.2	96.2	96.2	96.2
	Italy	Lombardy	52	100	100	100	96.2	96.2	96.2	96.2
	UK	Wales	336	99.4	97	96.1	95.8	95.8	95.5	95.5
	Italy	Tuscany	184	98.4	96.7	96.7	95.1	95.1	95.1	95.1
	Slovak		447	100	95.3	Not	Not recorded	Not	Not	94.6
	Republic					recorded		recorded	recorded	
	France	Paris	308	96.8	95.8	94.5	Not recorded	Not	Not	94.2
								recorded	recorded	
	Spain	ECEMC hospitals not reporting ETOPFA	557	95.5	93.7	93.7	93.7	93.7	93.7	93.7
	Czech		1,096	99.4	97.9	97.4	94.5	93.8	93.2	93.2
	Republic									
	Netherlands	EUROCAT Northern Netherlands	466	98.7	97.4	95.7	93.8	92.9	92.7	92.7
	Ukraine	OMNI-Net BD Program	191	99.5	96.3	94.2	92.7	91.6	91.6	91.6
	Mexico	RYVEMCE	1,124	94	91.4	91.4	91.4	91.4	91.4	91.1
	Sweden		2,972	98.4	96.4	95.2	92.8	92.1	90.9	90.9
	Colombia	Cali	11	100	100	100	90.9	90.9	90.9	90.9
	USA	Arkansas	498	97.4	94.6	92.6	90	89.2	89	89
	USA	Atlanta	392	96.7	92.9	89.8	87.8	87.5	87.2	87.2
	USA	Utah	699	95	91.6	90.1	87.6	86.7	86.4	86.4
	Argentina	RENAC	955	100	86.3	86.3	86.3	86.3	86.3	86.3
	Malta		34	100	94.1	91.2	85.3	85.3	85.3	85.3
	S-America	ECLAMC	2,594	90.4	86	83.2	82.8	82.8	82.8	82
	Mexico	Nuevo Léon	44	97.7	93.2	86.4	81.8	81.8	81.8	81.8
	Israel		54	100	94.4	92.6	83.3	83.3	81.5	81.5
	Total averages			98	95	93.8	91.5	91.3	91.1	91
Isolated	Spain_	ECEMC reporting ETOPFA	77	100	100	100	100	100	100	100
	Italy	Lombardy	37	100	100	100	100	100	100	100
	Malta		20	100	100	100	100	100	100	100
	Germany	Saxony-Anhalt	371	100	99.7	99.7	99.7	99.7	99.7	99.7
	France	Paris	254	99.6	99.6	99.6	Not recorded	Not recorded	Not recorded	99.6
	UK	Wales	253	100	99.6	99.6	99.6	99.6	99.6	99.6
	Spain_	ECEMC hospitals not reporting ETOPFA	388	99.5	99.5	99.5	99.5	99.5	99.5	99.5
	Slovak Republic		348	100	99.1	Not recorded	Not recorded	Not recorded	Not recorded	99.1
	Ukraine	OMNI-Net BD Program	156	100	100	99.4	98.7	98.7	98.7	98.7
	Netherlands	EUROCAT Northern Netherlands	391	99.7	99.2	99	98.7	98.5	98.5	98.5
	Sweden		2,437	99.8	99.5	99.3	98.7	98.6	97.6	97.6
	USA	Utah	511	99.6	98.6	98.6	97.7	97.5	97.5	97.5
	Mexico	RYVEMCE	878	98.1	97.4	97.4	97.4	97.4	97.4	97.2
	Argentina	RENAC	696	100	97.1	97.1	97.1	97.1	97.1	97.1
	S-America	ECLAMC	1,673	98.7	97.8	97	97	97	97	96.9
	Colombia	Cali	11	100	100	100	90.9	90.9	90.9	90.9
	Israel		45	100	93.3	91.1	88.9	88.9	88.9	88.9
	Isolated averages			99.7	98.9	98.6	97.8	97.8	97.7	97.7
Multiple Congenital Anomalies	Spain	ECEMC hospitals reporting ETOPFA	20	100	100	100	100	100	100	100
	UK	Wales	55	100	90.9	90.9	89.1	89.1	89.1	89.1
	Germany	Saxony-Anhalt	56	96.4	94.6	91.1	87.5	87.5	87.5	87.5
	Italy	Lombardy	15	100	100	100	86.7	86.7	86.7	86.7
	Spain	ECEMC not reporting ETOPFA	110	89.1	84.5	84.5	84.5	84.5	84.5	84.5
	Slovak Republic		90	100	85.6	Not recorded	Not recorded	Not recorded	Not recorded	84.4
	France	Paris	37	89.2	86.5	86.5	Not recorded	Not recorded	Not recorded	83.8
	Malta		11	100	90.9	81.8	81.8	81.8	81.8	81.8
	Netherlands	EUROCAT Northern Netherlands	35	94.3	94.3	85.7	80	80	80	80
	Ukraine	OMNI-Net BD Program	24	100	83.3	79.2	79.2	79.2	79.2	79.2
	USA	Utah	116	89.7	85.3	82.8	77.6	75	75	75
	Mexico	RYVEMCE	141	81.6	72.3	72.3	72.3	72.3	72.3	71.6
	Sweden		331	94	89.1	84	76.4	73.7	71.6	71.6
	Argentina	RENAC	232	100	59.1	59.1	59.1	59.1	59.1	59.1
	S-America	ECLAMC	921	75.5	64.6	58.1	57.1	57.1	57.1	54.8
	Israel		9	100	100	100	55.6	55.6	44.4	44.4
	MCA averages			94.35	86.32	83.84	78.68	78.35	77.52	77.1
Genetic / Chromosomal	Spain	ECEMC hospitals reporting ETOPFA	4	75	75	75	75	75	75	75
	UK	Wales	28	92.86	85.71	75	75	75	75	75
	Spain_	ECEMC hospitals not reporting ETOPFA	59	81.36	72.88	72.88	72.88	72.88	72.88	72.88
	Mexico	RYVEMCE	105	76.19	66.67	66.67	66.67	66.67	66.67	66.67
	Netherlands	EUROCAT Northern Netherlands	40	92.5	82.5	72.5	57.5	50	47.5	47.5
	Slovak Republic		13	100	61.54	Not recorded	Not recorded	Not recorded	Not recorded	46.15
	Germany	Saxony-Anhalt	16	87.5	68.75	56.25	43.75	43.75	43.75	43.75
	Sweden		204	89.22	71.57	65.2	49.02	44.61	42.16	42.16
	Argentina	RENAC	27	100	40.74	40.74	40.74	40.74	40.74	40.74
	France	Paris	17	70.59	58.82	35.29	Not recorded	Not recorded	Not recorded	35.29
	USA	Utah	72	70.83	51.39	41.67	31.94	29.17	26.39	26.39
	Czech Republic		33	93.94	72.73	66.67	39.39	33.33	24.24	24.24
	Ukraine	OMNI-Net BD Program	11	90.91	72.73	54.55	36.36	18.18	18.18	18.18
	Italy	Lombardy	1	100	100	100	0	0	0	0
	Malta		3	100	66.67	66.67	0	0	0	0
	Genetic/chromosomal averages			88.1	69.8	63.4	45.7	43.1	42	40.9

*Note*: Not all registries reported all deaths at all timepoints, data in this table reports deaths where data was returned. Where data were confirmed as not reported by a registry this have been presented in the table and the overall survival covers only the time period for which data were returned.

Abbreviations: CLP, Cleft lip with palate; ECEMC, Registry of the Spanish Collaborative Study of Congenital Malformations; ECLAMC, Latin American Collaborative Study of Congenital Malformations; ETOPFA, early termination of pregnancy for fetal anomaly; ICBDSR, International Clearing House for Birth defects Surveillance and Research; MCA, Multiple Congenital Anomalies; RENAC, National Network of Congenital Anomalies of Argentina; RYVEMCE, Mexican Registry and Epidemiological Surveillance of External Congenital Malformations; SMC, Soroka Medical Center; UK, United Kingdom; USA, United States of America.

## Data Availability

The data that support the findings of this study are available on request from the corresponding author. The data are not publicly available due to privacy or ethical restrictions.
